# Latest Innovations in the Treatment of Venous Disease

**DOI:** 10.3390/jcm7040077

**Published:** 2018-04-11

**Authors:** Robert R. Attaran

**Affiliations:** Cardiovascular Medicine, Yale University, 789 Howard Avenue, New Haven, CT 06519, USA; robert.attaran@yale.edu

**Keywords:** venous disease, venous insufficiency, venous reflux, deep vein thrombosis, post-thrombotic syndrome

## Abstract

Venous disease is more common than peripheral arterial disease. Pathophysiologically, venous disease can be associated with obstruction, reflux, or both. A common feature in chronic venous disease is ambulatory venous hypertension. Inflammatory and pro-thrombotic mechanisms can be activated. The current therapies, including compression, ablation, and recanalization are discussed.

## 1. Introduction

Venous disease is more common than peripheral arterial disease, affecting nearly 30 million in the United States of America (USA) alone [1,2,3,4]. Venous disease manifestations encompass spider veins, reticular and varicose veins, stasis dermatitis and pigmentation, lipodermatosclerosis, edema, and ulceration. Its prevalence increases with age, and it can manifest with debilitating symptoms, including leg heaviness, aching, fatigue, edema, and even ulceration [5,6]. Chronic venous disease has a large impact on quality of life and morbidity with annual costs of care estimated to exceed $3 billion in the United States [6,7].

Pathophysiologically, venous disease can be associated with obstruction, reflux, or both. Venous valves are designed to maintain antegrade flow through veins. Venous outflow obstruction or venous reflux can lead to ambulatory venous hypertension. This can lead to inflammation and edema [8]. Venous ulcers can affect at least 1–2% of the elderly population and carry a significant burden in terms of quality of life and health-care costs [3,9].

Veins have thinner media than arteries, and they possess valves. The thinner media allows for greater distensibility. A common feature in chronic venous disease is ambulatory venous hypertension. With standing, ankle venous pressures elevate in all individuals. The utilization of the so-called calf muscle pump in healthy individuals, however, can improve venous return and lower ankle venous pressure. In certain disease states, such as in muscle pump inefficiency, venous reflux, or obstruction, ankle venous pressure remains elevated. As leg elevation is not a practical solution, alternative therapies have been evaluated. Compression therapy, in some form or another, remains the gold standard treatment for chronic venous hypertension. Whilst compression therapy does not lower extremity venous pressure, it can reduce interstitial pooling. This in turn may improve tissue perfusion and serve an anti-inflammatory role [10].

Furthermore, human skin, compared to that of other upright animals, such as the giraffe or horse, is compliant. With standing, increased venous pressure can drive more fluid into the interstitium of humans [11,12].

The calf muscles, in particular the soleus, are thought to be primarily responsible for deep vein pump action [13,14]. This may explain why a sedentary lifestyle or neuromuscular degenerative disease can exacerbate venous stasis. Obesity, lower extremity joint deformation, and other osteoarticular pathologies may also contribute to muscle pump failure.

Elevated venous pressure may lead to maladaptive remodeling of the venous wall, leading to the development of enlarged and tortuous veins [15,16]. This process is associated with venous valve dysfunction [17].

Age, gender, pregnancy, weight, height, race, diet, bowel habits, occupation, posture, previous deep venous thrombosis, and genetics have all been proposed as predisposing factors for the formation of varicose veins [15,16,17,18,19]. It has been proposed that the predisposition to varicose veins is inherited in an autosomal dominant pattern with variable penetrance and with greater expression in the setting of hormonal factors, such as progesterone and estrogen, thus favoring disease prevalence in women [20].

## 2. Cellular and Inflammatory Basis

Matrix metalloproteinase proteins (MMPs) are significant factors in tissue remodeling and the promotion of the degradation of extracellular matrix (ECM) proteins [21]. Changes in MMP levels may alter vein wall structure and function [22]. MMP overexpression in venous ulcers is associated with poor healing [23].

Studies appear to suggest an increase in varicose veins of type I collagen content and a decrease in type III collagen content, the latter being a factor in venous elasticity [24,25]. Both elastin and laminin content are decreased in varicose veins [26]. In venous reflux disease, the veins do not demonstrate the same contractile response to agents that promote venoconstriction [27].

There is also an inflammatory component. Elevated levels of D-dimer, interleukin-6 (IL-6), interleukin-8 (IL-8), and C-reactive protein (CRP) levels were found within varicose vein blood in patients with venous insufficiency, compared with upper extremity blood samples [28,29]. Increased venous pressure is associated with increased leucocyte infiltration [30] and abnormal fibroblast function [31]. The increase in endoluminal venous pressure may be an important trigger not only for the release of pro-inflammatory but also prothrombotic factors [32]. The extravasation of proteins and iron may also be triggers of the inflammatory response. In a mouse model, dermal iron overload and its activation of macrophages was found to lower ulcer healing [33].

Activated macrophages and mast cells are found in the dermis of patients with chronic venous insufficiency (CVI) [34,35]. The presence and activity of mast cells may explain the pruritis seen in patients with venous insufficiency [34,35].

Clinically, edema, hyperpigmentation, lipodermatosclerosis, and venous ulceration are the sequelae of chronic venous hypertension.

## 3. Compression Therapy

Despite all the advances in the treatment of venous disease, compression therapy remains the cornerstone of therapy. An extensive review of the topic was published by Attaran et al. in 2017 [36]. External compression can improve venous return and lower interstitial edema [37,38]. Table 1 describes the grades of compression under American, German, and British standard classifications, as well as the author’s recommended indications [36].

Venous ulcer levels of inflammatory cytokines IL-1 and interferon-γ fall with compression therapy [39]. Compression with 30–40 mmHg in mainly C2–C3 venous disease (varicose veins or edema) can lower pain, pigmentation, and edema [40]. In venous ulcer disease (C6), there is data to support higher pressure (30–40 mmHg) compression versus lower for improved healing and prevention of recurrence [41]. However, some patients will find application of 30–40 mmHg compression stockings prohibitively challenging. Adherence rates can be poor [42]. In non-ulcer forms of venous disease (e.g., C2–C4), if patients cannot tolerate or apply the higher-pressure stockings, we recommend lower grade compression. Knee-high hosiery is typically more comfortable than thigh-high. There are donning devices available that can facilitate the application of stockings. Alternative designs that might be easier to apply, such as Velcro strap stockings, are also available. There is scant evidence that compression therapy improves procedural success after an ablative procedure. However, it may lower discomfort and edema [43].

## 4. Endovenous Laser Ablation

Thermal ablation for superficial truncal reflux has revolutionized vein therapies in the 21st century. Prior to it, surgical stripping and ligation were the standard of care, though selected patients may still be better treated with ligation (with or without phlebectomy). Thermal ablation can be performed with endovenous laser ablation (EVLA) or radiofrequency ablation (RFA).

EVLA was approved in the USA in 2001. The laser wavelength can target water or hemoglobin, leading to thermal injury to the target vein endothelium and its resultant occlusion. Wavelengths targeting water may require lower energy and lead to less potential thermal injury [44]. The EVLA fiber is a low-profile device that can be advanced over a wire and then activated and withdrawn slowly. Target veins can include an incompetent great saphenous vein (GSV), small saphenous vein (SSV), accessory, or perforator. Using ultrasound (US) guidance, a sheath is placed into the target vein, typically as downstream as feasible. The fiber tip, with US guidance, is placed at least 2 cm downstream from the deep vein junction. At the saphenofemoral junction (SFJ), it is also recommended to visualize and move the fiber downstream from the superficial epigastric vein confluence. After the careful administration of tumescent anesthesia, ablation can proceed. Some laser operators advise against manual external pressure applied on the vein during ablation, as it may lead to higher rates of pain and ecchymosis [45]. The use of a jacket tip and 1470-nm wavelength laser is associated with lower discomfort [46].

A registry of more than 1000 EVLA patients showed one-year occlusion rates of 93.1%, with sensory nerve injury of 2.7% [47]. A randomized comparison of EVLA versus surgery (SFJ ligation and GSV stripping) found higher rates of clinically visible recurrence with EVLA at 5 years. However, there was no difference in the clinical severity score or quality of life measures [48].

## 5. Radiofrequency Ablation

**Figure 1:**
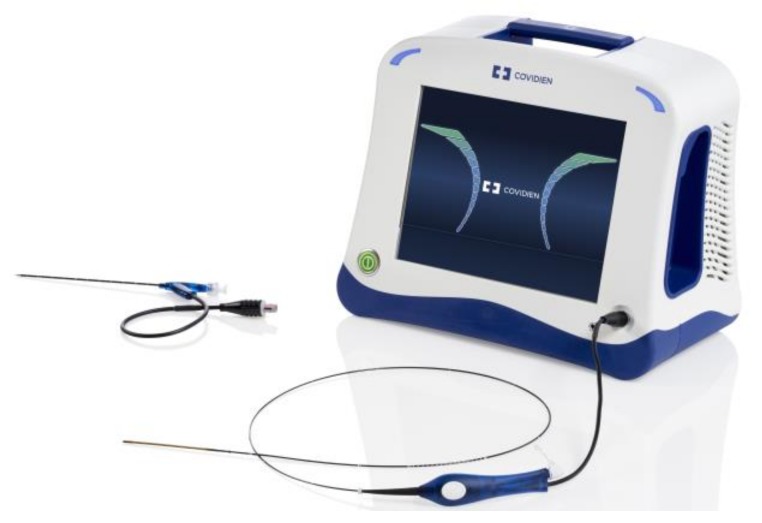
The ClosureFast radiofrequency ablation catheter.

Radiofrequency ablation (RFA) is another thermal-based technology to occlude saphenous veins. It has been approved in the USA for this indication since 1999. An electrode element is inserted into the target vein. The tissue acts as a resistor, and the molecules surrounding the element become excited and heat up. Thermal injury occurs within the vein lumen leading to thrombosis and eventual occlusion [49]. The current device used in the USA is the ClosureFast (Medtronic, Minneapolis, MN, USA) (Figure 1). Just as with EVLA, tumescent anesthesia is administered around the target vein. Tumescent anesthesia contains saline, lidocaine, epinephrine, and bicarbonate. The combination acts as a protective buffer and heat sink, as well as a local anesthetic. It will also protect surrounding structures from heat injury, including nerves, skin, and muscle. The presence of bicarbonate lowers discomfort from administration. Epinephrine increases venoconstriction allowing better endovenous contact with the heating element. It is important not to inject tumescent anesthetic directly into a vein as the aforementioned drugs will rapidly cycle into the cardiovascular system. Tumescent anesthesia must therefore always be administered under ultrasound guidance.

A ClosureFast device is available for both saphenous and perforator vein ablation. The current device for the saphenous veins has a lumen through which a wire can be advanced into the target vein. A wire is not routinely needed unless the catheter cannot traverse a tortuous segment. The device tip heats up to 120 °C for 20 s per cycle. The heating element is withdrawn after every cycle, and a new segment of vein is treated [50].

There is excellent outcome and follow-up data for RFA. Five-year prospective follow-up data on 295 GSVs showed an occlusion rate of 92% with only 2.7% of patients symptomatic [51]. A meta-analysis showed technical success rates of 88.7% for RFA and 84.8% for EVLA in treating GSVs [52]. Post-procedural pain and ecchymosis is likely higher with EVLA compared with RFA [53].

## 6. Endothermal Heat-Induced Thrombosis

A relatively rare but important complication of thermal saphenous vein ablation is endothermal heat-induced thrombosis (EHIT). The saphenous vein thromboses after thermal ablation. EHIT is the proximal propagation of thrombus into the deep venous system (femoral or popliteal vein). A review of randomized control trials of thermal ablation found EHIT rates to be <1% [54].

To achieve lower rates of EHIT, it is important during both EVLA and RFA to begin ablation at 2.5 cm or greater distal to the deep vein. It is this author’s opinion that ablation must be performed distal to the superficial epigastric vein. In addition to sparing the function of this vein, this will allow flow through the SFJ into the deep system, and this might lower risk of thrombus extension [55].

There are different grades of EHIT. Thrombus may extend up to the junction with the deep venous system (Grade I), extend into <50% of the deep vein (Grade II), extend >50% (Grade III), or completely occlude the deep venous system (Grade IV). Larger diameter GSVs (≥7.5 mm) may be a risk factor for EHIT. Risk of pulmonary embolism with EHIT is low or even negligible [56]. It is the opinion of this author that any EHIT extension into the deep system (i.e., Grade II or higher) should be treated with anticoagulants and a follow-up ultrasound study performed in 1–2 weeks. It is rare that anticoagulation is required beyond 30 days. However, there is inadequate data on the appropriate care of EHIT, and it is plausible that Grades II and III may resolve spontaneously.

## 7. Non-Thermal Techniques

Though thermal techniques have shown good efficacy for sealing incompetent veins, they have some shortcomings. They require the application of tumescent anesthesia, which involves additional needle insertions for its application. This leads to a prolonged procedure time and is usually the most painful part of the procedure for the patient. The heat energy can cause thermal injury, skin burns, or nerve injury if the tumescent anesthetic does not provide enough separation between the heating element and surrounding tissue. Furthermore, EHIT may result. Several non-thermal, non-tumescent (NTNT) techniques to eliminate axial reflux and varicose veins are now available for use. These currently include sclerotherapy, mechanochemical ablation (MOCA), and cyanoacrylate.

### 7.1. Sclerotherapy

Venous sclerotherapy refers to the intravenous injection of a substance that would bring about its chemical occlusion and obliteration. Various forms of venous sclerosing agents have been in use for centuries. In modern times, a number of sclerotherapy agents (e.g., sodium tetradecyl sulfate, polidocanol, hypertonic saline, and glycerin) have been used in different preparations (e.g., liquid and foam) [57,58,59]. Polidocanol and sodium tetradecyl sulfate (STS) belong to the detergent class.

The mixture of air with a sclerosant to inject into varicose veins was first described in the 1940s [60]. In 2001, Tessari et al. published their initial experience with a novel foam sclerosant and described a technique to “agitate” the sclerosant into a foam, using two syringes and a stop-cock [61]. A combination of the sclerosant and CO_2_ or air is drawn into the syringes and vigorously injected back and forth, creating a foam. Normally, sclerosants have the disadvantage of becoming diluted by the venous blood. The utility of the foam is its ability to displace more blood from the target vein lumen, allowing for more effective sclerosant delivery to the endothelium. A prospective multi-center study showed superior GSV reflux resolution rates with foam versus liquid sclerotherapy (69% vs. 27%) [62].

There is also a 1% polidocanol formulation named Varithena (BTG International, West Conshohocken, PA, USA) [63]. Varithena is FDA approved (since 2013) for the closure of visible varicose veins, as well as truncal incompetent veins. Its efficacy was demonstrated in VANISH-1 and -2 [64,65]. Saphenous injection of Varithena in the VANISH trials was complicated by deep vein thrombosis (DVT) in 4.7% of cases [64,65]. For injection of the Varithena polidocanol foam into the saphenous system, it is recommended to compress the SFJ or SPJ to prevent proximal migration. Ultrasound is used to visualize the distribution of foam.

Despite their potential for passage through the deep venous system and beyond, liquid sclerosants have demonstrated a good safety record [66,67]. They are a versatile tool and can be used to sclerose tiny vessels, such as telangiectasia, up to larger vessels, such as varicose veins.

Sclerosants used to treat GSV incompetence have shown varying results, with overall significant inferiority to thermal techniques, mechanochemical ablation (MOCA), or cyanoacrylate, in terms of long-term success rates [68,69]. Two studies comparing 1% vs. 3% foam sclerotherapy of the great saphenous vein showed no statistically significant difference in closure rates [70,71].

Complications of sclerotherapy include phlebitis and pigmentation (common), ulceration, and very rarely, neurologic sequelae, such as stroke. Sites of varicose vein sclerotherapy can trap so-called “coagulum”, which can be tender. Some operators drain these using a large needle or small blade.

There is little evidence to suggest that clinically significant right-to-left shunting through a patent foramen ovale occurs commonly with foam sclerotherapy [72]. It has been suggested that some of the migraine-like neurologic sequelae [73] may in fact be due to endothelin release.

DVT can also occur, particularly if large volumes of foam sclerosant are injected. To minimize risks of embolization or DVT, this author recommends using well-agitated foam with tiny bubbles (i.e., hard to visually discern) and small volume injections. When injecting a superficial vein, a slow and unforced injection is desirable. Displacement of blood in a visible varicose vein should be observed and the injection stopped when the blood has been displaced and the “leading edge” of the foam is no longer visible.

Accidental intra-arterial injection of sclerosant can lead to severe tissue necrosis and must be avoided with diligent technique [74]. There are case reports of the use of steroids and anti-coagulants to treat arterial injection of sclerosant [75].

### 7.2. Mechanochemical Ablation

**Figure 2:**
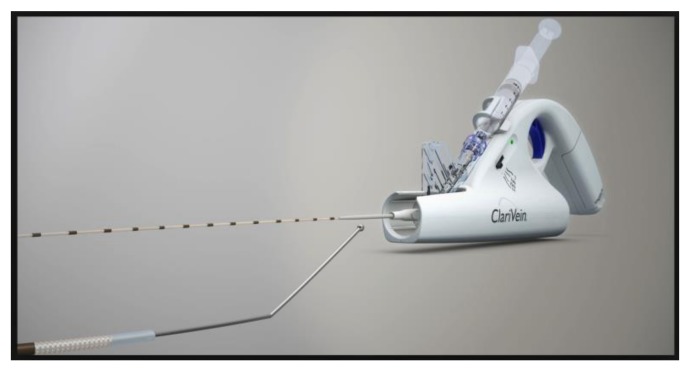
The ClariVein mechanochemical ablation catheter.

ClariVein (Vascular Insights, Quincy, MA, USA) is a system that utilizes so-called mechanochemical ablation (MOCA) to occlude a saphenous vein (Figure 2) [76]. A small, curved tip fiber is inserted into the vein, spun with a small electric motor at up to 3500 rpm, scratching the endothelial surface. At the same time, a small amount of sclerosant is injected through its rotating tip. During this process, the fiber is very slowly withdrawn. MOCA induces endothelial inflammation, thrombosis, and occlusion by combining mechanical injury and chemical irritation. The mechanical action of the spinning fiber is also thought to induce venoconstriction allowing for greater sclerosant contact with the endothelium. The device has a battery within its motorized handle. No generator is required. Similar to thermal ablation, under ultrasonic guidance, the device tip is positioned at least 2.5 cm from the SFJ before treatment is commenced. Mechanical rotation is initiated for at least 3 s together with slight withdrawal of the fiber without the administration of sclerosant, to induce venospasm and reduce entry of the sclerosant into the deep venous system. External pressure over the treated vein is recommended particularly for larger diameter GSVs. There is currently no consensus on the concentration of sclerosant to administer, but typical values are 1.5% STS administered at 0.1–0.2 mL/cm, above the knee [76,77].

Initial data has shown successful GSV occlusion rates with the ClariVein device, in the 94% plus range and comparable to RFA [76,78]. Also, lower procedural pain was noted compared to RFA [79,80,81]. Kim et al. [82] published two-year follow-up data on 65 patients who underwent MOCA for GSV reflux. Closure rates were 92%. Five patients had partial to complete recanalization. There was significant improvement in the venous clinical severity scores.

Complications from MOCA include DVT (very rare), phlebitis, and local hematoma [83]. The tip of the device can sometimes become trapped within the target vein (or a valve). Removal can sometimes require applied force and cause pain. In addition, it can be challenging to inject a steady stream of sclerosant through the device. In some venous segments, attempting to press the syringe plunger can be met with stiff resistance. If care is not exercised, a large amount of sclerosant may accidentally be injected when there is a sudden “give”.

### 7.3. Cyanoacrylate

**Figure 3:**
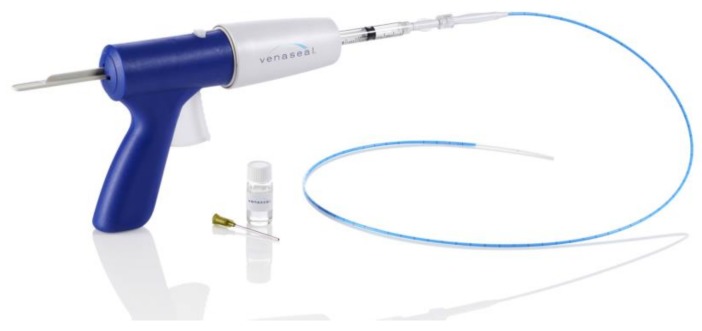
The VenaSeal cyanoacrylate delivery catheter.

Another available technology to seal the saphenous veins is cyanoacrylate adhesive (“superglue”) [84]. Various cyanoacrylate formulations have been used in medicine for decades. There are currently two cyanoacrylate vein adhesive devices available: VenaSeal (Medtronic, Minneapolis, MN, USA) (Figure 3) and VariClose (Bioas, Ankara, Turkey). Through a sheath, a delivery catheter is advanced into the saphenous vein. Through the catheter, the cyanoacrylate is carefully injected using a gun-handle mechanism. Within the GSV, typically a 5-cm distance is observed from the SFJ (for VenaSeal) to prevent glue migration into the deep system. After every administration using ultrasonic visualization, the catheter is withdrawn, and prolonged manual pressure is applied. After the entire desired length of vein has been treated, the fiber and sheath are removed. Compression or wraps are not necessary.

Almeida et al. reported 36-month follow-up data on the first-in-human cyanoacrylate trial. Of the 29 subjects available for follow-up, 94.7% showed saphenous vein occlusion rates. There was an improvement in the mean venous clinical severity score from 6.1 to 2.2 (*p* < 0.0001) [85].

VenaSeal was compared in a head-to-head fashion with RFA in the VeClose Study. From the 222 patients with GSV incompetence prospectively randomized to each treatment, 12-month follow-up data was available for 192 patients. Complete GSV occlusion was at 97.2% for cyanoacrylate and 97% for RFA. There were equivalent improvements in symptom scores [86].

A common complication of cyanoacrylate vein closure is phlebitis. This has been reported to occur in 11.4% of cases by Proebstle et al. [87] and 23.5% of cases by Park [88]. It typically resolves within 2 weeks.

## 8. Deep Vein Insufficiency

Deep venous insufficiency is a known cause of chronic venous insufficiency [89,90]. It can co-exist with venous outflow obstruction [91]. A common scenario is deep vein reflux after a DVT, which can contribute to the post-thrombotic syndrome (PTS) [92]. To restore some valvular function, several open surgical techniques have been described, including femoral to saphenous vein transposition [93], transplantation from the axillary or brachial vein [94,95], and neovalve formation [96]. An intriguing technique is neovalve formation as described by Maleti [96], wherein a new valve leaflet is dissected out and fashioned out of the venous endothelium. Maleti has utilized stiches between the flap and opposing venous wall to help maintain flap patency [97]. This technique appears to result in improved valvular competence upon follow-up. None of the aforementioned techniques are in prevalent use, however.

The first in-human trials are underway to evaluate BlueLeaf (InterVene, San Francisco, CA, USA), a catheter-based technology to create venous neovalves. Trials are expected to commence in the USA in late 2018. For now, however, a practical technique for the reconstruction or replacement of dysfunctional venous valves with sustainable results remains elusive.

## 9. Venous Obstruction

Venous outflow obstruction can be either primary (non-thrombotic) or secondary (post thrombotic) [91]. May–Thurner Syndrome is a condition where classically there is compression of the left common iliac vein between the right common iliac artery and the lumbar vertebrae [98,99]. Variants of it exist, affecting both iliac veins. Though often free of symptoms, May–Thurner Syndrome is thought to increase the risk of ipsilateral leg edema or DVT [100]. In the severest forms, venous collateral formation will be observed (e.g., via internal iliac veins or retroperitoneal/gonadal veins).

Imaging modalities for diagnosis include computed tomography or magnetic resonance venography. Invasive venography with intravascular ultrasound is, in this author’s opinion, the most sensitive means to diagnose venous stenosis and/or May–Thurner Syndrome.

The current standard treatment for May–Thurner Syndrome and/or iliac vein stenosis is angioplasty and stenting [101,102]. Stents are typically self-expanding and must withstand compressive forces. Intra-vascular ultrasound must be used for correct sizing. Undersized stents can increase the risk of stent migration into the inferior vena cava (IVC) or right ventricle.

The use of iliac intervention for the treatment of symptomatic iliac vein stenosis has shown favorable long-term patency, as well as improvement in symptoms including pain, edema, and ulcer healing [101]. Several new stent designs have been evaluated and are pending approval. While there is no strong clinical data on appropriate medical therapy after iliac intervention, many operators place the patient on anticoagulants for 3–6 months thereafter, particularly if the patient had a prior DVT. Post-procedural screening is strongly recommended. At our institution, we perform ultrasound studies every three months in the first year and less frequently thereafter, to ensure stent patency. Intervention on a chronically occluded stent can be technically difficult. There is further discussion of venous obstruction in Section 11.

## 10. Acute Deep Vein Thrombosis

The commonest site for DVT is the leg. It can be complicated by pulmonary embolism (PE). Its incidence is high, particularly in individuals above age 50 [103]. In addition to significant initial morbidity, DVT recurrence rates are approximately 30% after 8–10 years [104,105]. Risk factors for DVT/PE include hypercoagulable disorders [106,107,108], immobility [109], surgery [110,111], malignancy [112], infection [113], pregnancy [114], hormone replacement therapy [115], oral contraception [116], intravenous drug use and central lines [117,118,119], smoking, and obesity [120,121], among others. Typically, its etiology is multifactorial.

The interaction of thrombus with venous endothelium is thought to trigger a strong inflammatory response and injury [122], clinically referred to as thrombophlebitis. This is followed by venous valvular destruction.

Meissner et al.’s data on patients receiving prompt thrombolysis and thrombus removal in acute DVT suggests that it helps preserve valvular function [123]. Two randomized trials in catheter-directed therapy for DVT merit mention. The CaVenT Trial randomized patients with an acute iliofemoral DVT to anticoagulation alone (control) versus anticoagulation plus catheter-directed thrombolysis. The catheter in question was merely a perfusion catheter rather than a mechanical device. Five-year follow-up data was available for 176 patients, demonstrating that 71% of the control group developed PTS versus only 43% of the catheter thrombolysis group (*p* < 0.0001, NNT = 4) [124].

The much larger ATTRACT Trial (*n* = 692) prospectively randomized patients with both femoral and iliac acute DVT to anticoagulation alone (control) versus anticoagulation plus catheter thrombolysis therapy [125]. Most patients in the catheter group also underwent an additional mechanical procedure, such as balloon venoplasty, maceration, or stent placement. The primary end-point of PTS at two years was not significantly different between the two arms (48% in the control group and 47% in the catheter group). No significant difference in the rate of recurrent venous thromboembolism was seen. Also, there was more major bleeding in the catheter therapy group (1.7% vs. 0.3% (*p* = 0.049)). However, mean Villalta and VCSS scores were lower in the catheter-directed group, and there were lower rates of moderate-severe PTS (17.9% vs. 23.5% in controls (*p* = 0.035)). It has been difficult to reconcile the divergent findings of CaVenT and ATTRACT. It remains to be seen which type of patient and which location of DVT might benefit more from catheter thrombolysis of DVT as opposed to anticoagulation alone.

In rare cases, DVT can be complicated by phlegmasia cerulea dolens. Extensive edema can compromise arterial inflow in addition to venous outflow. Compartment syndrome and even limb loss can follow. There is general consensus that phlegmasia patients should be treated with (catheter) thrombolysis and angioplasty.

To date, anticoagulation remains the standard of care for the treatment of DVT. There are a number of societal guidelines for the choice and duration of anticoagulant, depending on the setting. The 2016 American College of Chest Physicians Guidelines, for instance, make several recommendations [126]. For venous thromboembolism (VTE) without cancer, a direct oral anticoagulant is recommended for at least three months. In the setting of cancer, low molecular weight heparins are recommended for at least three months, and patients should receive extended anticoagulation if they do not have a high bleed risk.

Recurrence of thromboembolism, however, remains a concern among patients who discontinue anticoagulants. The potential utility of prolonged anticoagulation in patients with a history of VTE was evaluated by the well-designed EINSTEIN CHOICE Trial [127]. Patients (*n* = 3396) were prospectively randomized to receive aspirin 100 mg vs. rivaroxaban 20 mg vs. rivaroxaban 10 mg, daily. At median follow-up of almost one year, rivaroxaban at either dose was superior to aspirin in preventing recurrent VTE (which occurred in 4.4% of the aspirin group, 1.2% of the rivaroxaban 10 mg group, and 1.4% of the rivaroxaban 20 mg group, *p* < 0.001). Rates of major bleeding were not significantly different.

## 11. Chronic Deep Vein Thrombosis and the Post-Thrombotic Syndrome

Post-thrombotic syndrome (PTS) is a chronic, debilitating condition that can affect the affected leg following DVT. It can affect upwards of 40% of individuals following DVT [125]. Visually, there can be hyperpigmentation, edema, lipodermatosclerosis, and ulceration. Symptomatically, there can be significant misery and pain, not to mention time lost from work [128].

DVTs involving the common femoral or iliac veins are more likely to lead to PTS, as are recurrent DVTs [129]. One diagnostic scoring system for PTS is the Villalta score, which includes five patient-reported symptoms and six clinician-reported features [130].

In a patient with PTS, the “open vein” theory suggests that improved venous outflow may alleviate some of the symptoms. Even if the valvular incompetence cannot be treated, the effective outflow stenosis that may be pre-existing (e.g., May–Thurner syndrome) or created by the synechiae and scarred veins may be amenable to intervention. There has been a significant recent increase in endovascular deep vein interventions involving stent implantation in the iliac veins (or IVC) and angioplasty in more distal veins [131].

It is recommended to obtain venous access distal to the area of interest. Suspected iliac vein pathology should ideally be approached from the popliteal or distal common femoral vein. Ankle vein access is recommended for suspected popliteal vein disease. In some cases, the jugular vein can be used either as the primary approach site or to support a distal approach. The use of ultrasonic guidance for access is strongly recommended.

Venous chronic total occlusions can be more challenging to cross than arterial occlusions. Most operators favor an 0.035 Glidewire advanced through a support catheter. As the occlusions can be hard in texture, some operators resort to off-label sharp recanalization techniques, utilizing a trocar from a transjugular intrahepatic portosystemic shunt kit. Once the occlusion has been crossed, a stiffer and more supportive 0.035 wire can be placed, and balloon inflations performed. Another technique that can improve procedural success in chronic occlusions is to infuse low dose thrombolytics intravenously from a site upstream (distal) to the occlusion over approximately 24 h. Venography is repeated the following day. In many cases, “small channels” will become apparent (“the string sign”) that will render wiring through the occlusion easier [101,132].

The ACCESS PTS Study evaluated the EKOS Catheter, an ultrasonic thrombolysis catheter, followed by balloon angioplasty, among patients with a history of prior iliofemoral DVT and PTS (Villalta score ≥8). The results of this single arm study were presented by Garcia at the Society of Vascular Medicine Scientific Sessions in June 2017. There was a reduction in Villalta scores of 34% at 30 days and a significant rise in quality of life scores. The biggest limitation of this study was its lack of controls.

## 12. Conclusions

Venous disease is very common and can be associated with obstruction and/or reflux. Patients can be symptomatic and, in some cases, debilitated. Compression therapy remains the mainstay of therapy though adherence can be poor. Compression wear can be difficult to apply and uncomfortable. Several minimally invasive catheter-based techniques have been developed to treat both venous reflux and obstruction, which can relieve some of the symptoms. They are in general safe and well-tolerated. Some of the many unanswered questions in the management of venous disease pertain to the optimal management of patients with DVT and post-thrombotic syndrome.

## Figures and Tables

**Table 1:** United States, British, and German standards for compression bandages in addition to recommended indications.

United States	German Standard	British Standard	Pressure (mmHg)	Recommended Usage
Light	KK1	3A	<20	Mild C1–3 disease, or unable to tolerate higher grade
Class I	KK2	3B	21–30	C1–C3 disease
Class II	KK3	3C	31–40	More symptomatic C2–C3 disease, C4 or higher, PTS
Class III	KK4	3D	>40	C5–6 disease (if not responding to lower grades)

The pressure range for the German Standard is different (KK1 = 18–21, KK2 = 23–32, KK3 = 34–46, and KK4 ≥ 49 mmHg). C1: Spider or reticular veins, C2: varicose veins, C3: edema, C4: lipodermatosclerosis, pigmentation, eczema, or atrophie blanche, C5: healed venous ulcer, C6: active venous ulcer. PTS: Post-thrombotic Syndrome.
